# The Value of Auditing the Frequency of Inhalational Anesthetics With a Combination of Very Low Bispectral Index and High Fraction of the Age-Adjusted Minimum Alveolar Concentration

**DOI:** 10.7759/cureus.75036

**Published:** 2024-12-03

**Authors:** Franklin Dexter, Richard H Epstein, Anil A Marian

**Affiliations:** 1 Anesthesia, University of Iowa, Iowa City, USA; 2 Anesthesiology, Miller School of Medicine, University of Miami, Miami, USA

**Keywords:** anesthesia information management system (aims), bispectral index monitoring, general anesthesia practice, inhalational anesthesia, minimum alveolar concentration (mac), prolonged tracheal extubation, retrospective cohort study

## Abstract

Background: Previously, a depth of anesthesia bispectral index (BIS™) <45 was considered low* *and found to have no clinical benefit. A BIS <35 was considered very low and was not only without evident clinical benefit but also associated with a greater risk of postoperative delirium. We considered the association between BIS and the anesthetic dose of inhalational agents, quantified using the minimum alveolar concentration (MAC) fraction, which was the patient’s end-tidal inhalational agent concentration divided by the agent’s altitude- and age-adjusted minimum alveolar percentage concentration. The MAC fraction was displayed on the anesthesia machine. When the MAC fraction >1.0 and the BIS <35, it implies that the inhalational anesthetic agent concentration can be reduced without harmful clinical effects. We hypothesized a substantive percentage of cases (>10%) have long (≥15 minutes) periods having the combination of MAC fraction >1.0 and BIS <35.

Methods: The retrospective database cohort study included N = 8,566 cases from September 13, 2016, to August 12, 2024 that met the following criteria: (1) ≥100 minutes of BIS monitoring (BIS minutes); (2) use of general anesthesia; (3) tracheal intubation and extubation performed in the operating room where the anesthetic was administered; and (4) absence of prone positioning. The latter three were characteristics of studies examining prolonged extubation, defined as ≥15 minutes from the end of surgery. From the *N* = 8,566 cases studied, 1,862,022 BIS minutes were automatically recorded in the electronic health record. Comparisons were made with the matching MAC fractions. A Clopper-Pearson two-sided exact 95% confidence interval was calculated for the planned primary endpoint, the percentage of cases with ≥15 minutes wherein simultaneously the BIS <35 and MAC fraction >1.0. Post hoc, we added a 97.5% confidence interval for the incidence with ≥30 minutes to compare with 10%.

Results: Among the 8,566 cases with ≥100 minutes of BIS monitor use, 29.5% (2,527) had prolonged extubation. There were contemporaneously 152,443 other cases without BIS monitoring. Those other cases had a nearly identical incidence of prolonged extubations (29.3%, 44,675). A total of 375 distinct anesthesia practitioners used the BIS monitor during the 8,566 cases, with each contributing, on average, only 0.27% (standard deviation [SD] = 0.33%) of anesthetic minutes. Among the *N* = 7,031 cases with BIS measured when the MAC fraction ≥0.6, 25% (1,780/7,031) had ≥15 minutes of very low BIS and MAC fraction >1.0. The 95% confidence interval was 24% to 26%. Being considerably larger than 10% (*P* < 0.0001), post hoc, we repeated the calculations using the threshold of ≥30 minutes of very low BIS and MAC fraction >1.0. The estimated incidence was 15%, with a 97.5% confidence interval of 14% to 16%, also significantly exceeding 10% (*P* < 0.0001).

Conclusions: If department practices are such that processed electroencephalographic monitoring (e.g., BIS) is commonly used during general anesthesia to assess anesthetic depth, our results recommend auditing the volatile agent concentration and the corresponding anesthetic depth index, as we did in this study. We recommend that feedback and education regarding suitable titration of anesthetic agent concentrations relative to the index should be provided at the departmental level.

## Introduction

We performed multiple studies to understand prolonged extubation times [1-5], defined as an interval from the end of surgery to tracheal extubation of 15 minutes or longer [6-8]. In our recently published prospective observational study, anesthesia providers were asked, when surgical closure began, for their target minimum alveolar concentration (MAC) fraction when the surgical drapes were lowered [4]. By *MAC fraction*, we refer to the patient’s end-tidal inhalational agent concentration divided by the agent’s altitude- and age-adjusted minimum alveolar percentage concentration [4,5,9,10]. This value is displayed on the anesthesia machine. Based on population pharmacokinetics and pharmacodynamics, MAC fractions between 0.4 and 0.5 would be a suitable target at the end of surgery [4,11]. However, among 56 patients, each with a different anesthesia provider, only 32% had a value within this range, and among practitioners, 13 goals were <0.4 and 25 goals were >0.5 [4]. This result was important because MAC fractions >0.4 at the end of surgery caused prolonged extubations (odds ratio 2.66, *P* < 0.0001) [5].

These findings of high variability in both the target and actual MAC fraction at the end of surgery raise the possibility of similar behaviors during surgery. Because our hospital’s regularly used device to assess depth of anesthesia is the bispectral index (BIS™) monitoring system (Medtronic, Minneapolis, MN), we focus on BIS, a unitless scale from 0 to 100. Previously, BIS <45 was considered *low* and was found to be without clinical benefit [12]. BIS <35 was considered very low [13] and was not only without evident clinical benefit [13] but also associated with a greater risk of postoperative delirium [14,15]. When the MAC fraction >1.0 and the BIS <35, the implication is that the inhalational anesthetic agent concentration can be lowered without harmful clinical effects. We hypothesized that there would be a substantive percentage of cases (>10%) with long (≥15 minutes) periods having the combination of MAC fraction >1.0 and BIS <35. If the hypothesis were not rejected, this would show that our department and others should use their electronic health records to check and provide departmental feedback regarding the appropriate titration of anesthetic dose when BIS monitoring is used.

## Materials and methods

Our retrospective cohort study (#202408389) was determined by the University of Iowa Institutional Review Board on August 22, 2024, not to meet the regulatory definition of human subjects research.

The retrospective database cohort study included N = 8,566 cases from September 13, 2016, to August 12, 2024 that met the following criteria: (1) ≥100 minutes of BIS monitoring (BIS minutes); (2) use of general anesthesia; (3) tracheal intubation and extubation performed in the operating room where the anesthetic was administered; and (4) absence of prone positioning [1,2,5]. The latter three were characteristics of studies examining prolonged extubation, defined as ≥15 minutes from the end of surgery. Detailed characteristics of numerous variables describing the underlying patient population, surgeons, and procedures are outlined in studies by Dexter et al. [2,5].

As defined earlier, *MAC fraction* refers to end-tidal inhalational agent concentration divided by the agent's altitude- and age-adjusted minimum alveolar concentration [5,9,10]. The altitude- and age-adjusted minimum alveolar concentration is a property of the inhalational agent obtained from earlier studies [9,10], while the MAC fraction is the dose reported as a fraction [4,5]. The MAC fraction is a summation of values among agents (e.g., if isoflurane was used for part of a case and desflurane later during the case) [4,5]. The study hospital's altitude was 204 m above sea level, resulting in an altitude adjustment of 0.977 times the measured concentration [5]. Age had a substantial effect, greater for individuals under 40 years and less for those over 40 years [5,10]. For example, for 40- versus 75-year-old patients at the hospital, the minimum overall mean end-tidal concentration of sevoflurane studied would be 1.3% and 0.9%, respectively [5,10]. At the study hospital, all anesthesia machines displayed the end-tidal agent concentration, which was recorded automatically in the health record [5]. The anesthesia machines also displayed the MAC fraction. Propofol, nitrous oxide, and other medications lower the BIS for given MAC fractions. In our study, we deliberately ignored the potential effects of these adjuvant mediations to underestimate the minutes with the combination of deep anesthesia and high anesthetic dose (i.e., to reduce the chance of the null hypothesis being rejected). We deliberately did not examine the association of BIS and MAC fraction because equal MAC fractions of different inhalational agents resulted in different BIS values [16,17].

From the *N *= 8,566 cases studied, 1,862,022 BIS minutes were automatically recorded in the electronic health record. The blinded case identifier and date/time were used for each minute to find the matching end-tidal concentrations of inhalational anesthetic(s). Then, the matching MAC fraction of the minute was calculated. Multiple sensitivity analyses were performed with different thresholds for cases with low BIS and lengthy (e.g., ≥15 and ≥30 minutes) periods of MAC fraction ≥0.6 or >1.0. A Clopper-Pearson two-sided exact 95% confidence interval was calculated for the planned primary endpoint, the percentage of cases with ≥15 minutes wherein simultaneously the BIS <35 and MAC fraction >1.0. We included all available data and thus did not perform a power analysis beforehand. We would consider the sample size sufficient if the confidence interval ruled out a clinically and managerially important 10% incidence of ≥15 minutes with very low BIS (<35) and MAC fraction >1.0. Post hoc, we added a 97.5% confidence interval for the incidence with ≥30 minutes to compare with 10%. The interval was 97.5%, reflecting adjustment for a second comparison. We used conservative (i.e., wide) Clopper-Pearson confidence intervals and selected two-sided intervals, even though the hypotheses were one-sided, to bias the analysis against rejecting the null hypothesis. Incidences were compared with 10% using two-sided exact binomial tests.

## Results

Between September 13, 2016, and August 12, 2024, there were 8,566 cases with ≥100 minutes of use of a BIS monitor (Table 1). Of those cases, 29.5% (2,527) had prolonged extubation. Over the same period, there were 152,443 other cases without BIS monitoring, without prone positioning, and with tracheal intubation in the operating room. Those other cases had a nearly identical incidence of prolonged extubations (29.3%, 44,675). Cases where BIS was measured for ≥100 minutes increased progressively from 126 in 2018 to 3,439 in 2023, with a projection of 4,018 in 2024. Sevoflurane was the predominant inhalational anesthetic, with negligible use of desflurane (Table 1). Neuromuscular reversal was used for 16% (1,393) cases. The patients were classified according to the American Society of Anesthesiologists Physical Status as follows: 50% (4,259) were classified as 1-2, 41% (3,742) as 3, and 9% (835) as 4-5. A total of 375 distinct anesthesia practitioners used the BIS monitor during the 8,566 cases, with each contributing, on average, only 0.27% (standard deviation [SD] = 0.33%) of the anesthetic minutes. The maximum contribution by a single practitioner was only 2.1% of minutes (i.e., our results represent the cumulative effect of hundreds of individuals). The studied cases involved 240 distinct surgeons, each contributing a mean of 0.42% of the cases (SD = 0.58%), with a maximum contribution of 3.9% of the total anesthesia minutes.

**Table 1:** Characteristics of bispectral index (BIS) observations, cases, and patients, stratified based on the age‑adjusted minimum alveolar concentration (MAC fraction) at each BIS minute. ^a^There were 3.1% (227/8566) patients aged <18 years. ^b^The mean isoflurane MAC fraction can equal 0.11 among minutes with MAC fraction ≥ 0.6 because the anesthesia practitioner switched from one agent to another. ^c^The case date is reported as a fraction to show the increases in BIS use. Successive years (2017-2023) had 147, 126, 216, 398, 627, 1,135, and 3,439 cases with BIS. There were 2,433 cases in 2024 through July (i.e., projected >4,000 annually). N/A, not applicable; SD, standard deviation

Variable	Cases	Mean	SD	Median	25th percentile	75th percentile
Patient age (years)	8,566	55.01	18.38	58.00	42.00^a^	69.00
BIS minutes per case	8,566	216.82	112.10	183.00	138.00	258.00
BIS minutes per case as a proportion of operating room time	8,562	0.76	0.12	0.78	0.70	0.85
BIS minutes per case with MAC fraction ≥0.6	8,566	142.92	119.10	133.00	44.00	201.00
BIS mean among minutes with MAC fraction ≥0.6	7,031	42.63	8.24	42.40	37.20	47.91
BIS minutes per case with MAC fraction >1.0	8,566	59.75	81.48	22.00	0.00	98.00
BIS mean among minutes with MAC fraction >1.0	5,843	41.09	8.93	40.96	35.30	46.53
Mean MAC fraction among minutes with MAC fraction ≥0.6	7,031	0.95	0.16	0.94	0.83	1.06
Mean MAC fraction among minutes with MAC fraction >1.0	5,843	1.14	0.16	1.12	1.07	1.18
Desflurane mean MAC fraction among minutes with MAC fraction ≥0.6	7,031	0.00	0.05	0.00	0.00	0.00
Isoflurane mean MAC fraction among minutes with MAC fraction ≥0.6	7,031	0.11^b^	0.27	0.00	0.00	0.00
Sevoflurane mean MAC fraction among minutes with MAC fraction ≥0.6	7,031	0.84	0.35	0.93	0.77	1.06
Date of the case, reported as a fraction of the study interval^c^	8,566	0.80	N/A	0.86	0.76	0.94
Nitrous oxide, expressed as a proportion of the fresh gas flow during the case	8,566	0.0045	0.0225	0	0	0
Midazolam (mg)	8,566	0.8	2.6	0.0	0.0	1.0
Propofol (mg)	8,566	167	412	0	0	200
Ketamine (mg)	8,566	7.1	22.2	0.0	0.0	0.0
Remifentanil (mcg)	8,566	61	1788	0	0	0

The MAC fraction was compared with 0.6 and 1.0 for each minute of deep anesthetic. Of the total cases, 46% (3,212) had ≥15 minutes of very low BIS (<35) and MAC fraction ≥0.6 (Table 2). Among the cases with BIS measured while MAC fraction ≥0.6, there were 25% (1780/7031) of cases with ≥15 minutes of very low BIS and MAC fraction >1.0. The 95% confidence interval was 24% to 26%. Being considerably larger than 10% (*P* < 0.0001), post hoc, we repeated the calculation using the threshold of ≥30 minutes of very low BIS and MAC fraction >1.0. The estimated incidence was 15%, with a 97.5% confidence interval of 14% to 16%, also significantly exceeding 10% (*P* < 0.0001).

**Table 2:** Incidences of cases with very low bispectral index (BIS) and large (>1.0) MAC fraction for lengthy periods (≥15 minutes) and several sensitivity analyses. The entries are reported as the percentage (numerator/denominator).

Percentage of cases meeting the criteria	Minutes with BIS <35 and MAC fraction ≥0.6	Minutes with BIS <35 and MAC fraction >1.0
≥10 minutes, added as sensitivity analysis	52% (3,691/7,031)	31% (2,205/7,031)
≥15 minutes, the planned primary endpoint	46% (3,212/7,031)	25% (1,780/7,031)
≥30 minutes, added after seeing the result of the planned analysis, as explained in the last paragraph of Methods	32% (2,276/7,031)	15% (1,077/7,031)

## Discussion

When inhalational anesthetics are being administered (i.e., with MAC displayed continuously on the anesthesia machine), patient outcomes are not improved by running the BIS below the recommended lower limit for general anesthesia (i.e., 40) [12,13]. Furthermore, very low BIS (<35) considered in this study is potentially harmful, as it is associated with an increased incidence of postoperative delirium [14,15]. BIS monitoring does not reduce the incidence of prolonged extubations when controlling for the MAC fraction at the end of surgery (odds ratio 0.94, 99% confidence interval 0.80-1.10, *P *= 0.89) [5]. Nevertheless, we only included cases with many minutes of BIS (≥100) and limited our consideration to the combination of BIS <35 and MAC fraction >1.0 for ≥15 minutes. Those conditions were present in 25% of the cases, with a lower two-sided 95% confidence limit of 24%. Importantly, these conditions were also present for >10% of cases for ≥30 minutes, with the lower 97.5% confidence limit on the incidence being 14%.

Our results imply that departments with a substantive incidence of BIS monitoring during general anesthesia should audit such use for departmental feedback and education about the titration of volatile agent concentrations to the BIS and appropriate use of the BIS. For example, during cases where the MAC fraction is planned to be maintained considerably >1.0 to prevent movement without using neuromuscular blockade, the expense of the BIS sensor may be unwarranted. Likewise, during cases where the MAC fraction is maintained >1.0 to control hemodynamics, the expense of the BIS sensor also seems unwarranted. Departmental feedback and education are suitable, as opposed to individual feedback, because none of the hundreds of anesthesia practitioners using the BIS monitor accounted for even 1.0% of the inappropriate BIS minutes (i.e., high MAC and very low BIS). Furthermore, the cases with deep anesthesia and MAC fraction >1.0% were <1.1% of cases with general anesthesia and tracheal intubation (1780/160,999). Thus, individual retrospective feedback would unlikely be effective because of a small fraction of each practitioner's cases. The departmental feedback can focus on the cost of the BIS sensors (i.e., do not apply them and then functionally ignore the values when dosing inhalational anesthetic). Departmental feedback can also focus on inhalational agents' cost and environmental effect (e.g., if using the BIS monitor, reduce the MAC fraction when the BIS is low, thereby reducing inhalational agent consumption) [18]. Until additional randomized trials provide greater insight into the impact of very low BIS (<35) with high anesthetic dose (MAC fraction >1.0) [12-15], available data do not support feedback based on the patient benefit of changing care.

Although our results were limited to the behavior of the anesthesia practitioners at a single department, there are multiple reasons to expect the generalizability of our results to other departments. First, 375 distinct anesthesia practitioners were studied. Second, results were as hypothesized based on a prospective observational study of 56 providers finishing anesthetics, wherein 68% (38/56) reported goal MAC fraction <0.4 or >0.5 for surgical drapes down (see Introduction) [4]. In the earlier study, even if the anesthesia practitioners had associated surgical stimulation with patients' physiological responses at different vaporizer MAC settings, the dose-response for awakening did not match responsiveness during induction and maintenance because of substantial pharmacodynamic hysteresis [11]. Our studied anesthesia practitioners' limited pharmacokinetic/ pharmacodynamic knowledge is disappointing but reflects reality [4]. Third, we only considered the MAC fraction of volatile anesthetics, although other medications increase the depth of anesthesia and reduce the BIS (e.g., propofol and nitrous oxide, Table 1). Thus, we deliberately underestimated the percentage of cases with ≥15 minutes of BIS <35 and MAC fraction >1.0. That is, an even greater fraction of anesthetics has inappropriate combinations of the two values than we report. Fourth, the direct costs of inhalational agents have progressively decreased over the past decades (e.g., sevoflurane is available in generic form). Therefore, the relative cost of the BIS sensor can be substantial, making the choice of its use important.

## Conclusions

A departmental audit of electronically recorded BIS monitor data and inhalational agent concentrations revealed that a quarter of the cases had ≥15 minutes of very low BIS and a MAC fraction >1.0. Furthermore, over 10% of cases had ≥30 minutes of very low BIS and MAC fraction >1.0. If departmental practices involve the routine use of processed electroencephalographic monitoring (e.g., BIS) during general anesthesia to assess anesthetic depth, our results suggest auditing both the volatile agent concentration and the corresponding anesthetic depth index, as we did in this study. We recommend that feedback and education regarding suitable titration of anesthetic agent concentrations relative to the index should be provided at the departmental level.
